# Inferior Mesenteric Vein-Caval Shunt for Extrahepatic Portal Venous Obstruction With Portal Cavernomatous Cholangiopathy: A Hobson’s Choice

**DOI:** 10.7759/cureus.73780

**Published:** 2024-11-15

**Authors:** Kaushal Rathore, Peeyush Varshney, Vaibhav K Varshney, Lokesh Agarwal, Binit Sureka

**Affiliations:** 1 Surgical Gastroenterology, All India Institute of Medical Sciences, Jodhpur, Jodhpur, IND; 2 Diagnostic and Interventional Radiology, All India Institute of Medical Sciences, Jodhpur, Jodhpur, IND

**Keywords:** colopathy, extra-hepatic portal hypertension, portal cavernomatous cholangiopathy, splenectomy, uncommon shunt

## Abstract

Proximal splenorenal shunt is the most commonly performed shunt in patients with extrahepatic portal venous obstruction (EHPVO). Sometimes, due to various anatomical and intraoperative factors, other rarely used shunts may be required. We present the case of a 27-year-old male who was diagnosed with EHPVO with complicated portal cavernomatous cholangiopathy. He had thrombosis of the entire extrahepatic portal vein, splenic vein, and superior mesenteric vein. An unconventional side-to-side inferior mesenteric vein (IMV) to inferior vena cava (IVC) shunt was performed. The patient had a chyle leak in the postoperative period, which was managed conservatively. Sixteen months after shunt surgery, the patient had no further bleeding, with a resolution of cholangiopathy, and is currently without any endobiliary stent. The IMV-caval shunt is a feasible and safe makeshift shunt with a good long-term outcome in the absence of a shuntable splenic vein.

## Introduction

Non-cirrhotic portal hypertension (NCPH) is portal hypertension with normal liver function; it is mainly due to non-cirrhotic portal fibrosis (NCPF) and extrahepatic portal venous obstruction (EHPVO). Almost 70% of upper GI bleeding in children and young adults in India is caused by EHPVO [1], which is due to congenital vascular malformation or acquired portal venous thrombosis (PVT) with a prior history of umbilical vein catheterization, trauma, surgery, or peritonitis in 25% of patients [2]. Over time, in order to maintain hepato-petal flow, there is the formation of collaterals, “the characteristic portal cavernoma formation.” Nowadays, endoscopic treatment is preferred for acute control of bleeding and prevention of recurrence of bleed [3,4], but single-time intervention in the form of shunt surgery can be a useful alternative as these patients have a normal liver function, leading to low rates of decompensation post-surgery [2]. The most common presentation in patients with EHPVO is GI bleed [1]. Indications for surgery include mainly massive painful splenomegaly, hypersplenism, growth failure, colopathy, and symptomatic portal cavernomatous cholangiopathy (PCC). Splenectomy with devascularization can take care of GI bleeding [5], but in the cases of biliopathy, colopathy, and severe portal gastropathy, shunt is usually required. A proximal splenorenal shunt (PSRS) is one of the most commonly performed procedures in patients with EHPVO, but in cases of anatomical variations in splenic/renal vein or their thrombosis, these shunts are not feasible; in such cases, alternatives like spleno-adrenal, superior mesenteric vein (SMV)-caval, and inferior mesenteric vein (IMV)-caval shunting can be useful alternatives. Although IMV-caval shunt has been previously described in cirrhotic cases, literature on its use in NCPH is sparse. We report an IMV-caval shunt in a patient with EHPVO with a non-shuntable splenic vein.

## Case presentation

**Figure 1:**
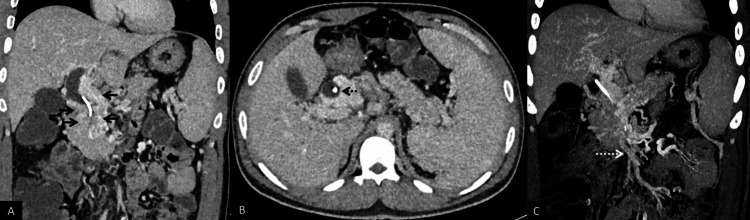
Preoperative CT images (A) Coronal reformatted CT image showing main portal vein replaced by pericholedochal venous collaterals of Petren (arrows) with non-visualization of splenic and superior mesenteric vein. (B) Axial CT image showing dilated common bile duct with stent in situ (dashed arrow) suggesting portal cavernomatous cholangiopathy (PCC). (C) Coronal maximum intensity projection (MIP) image showing patent inferior mesenteric vein (IMV) (dashed white arrow).

A 27-year-old male with no prior comorbidity presented to us with chief complaints of pain in the left upper abdomen, recurrent upper respiratory tract infections for the last three to four years, and one episode of GI bleeding six months back. He was evaluated elsewhere and was diagnosed with EHPVO with portal hypertension. Upper GI endoscopy revealed large esophageal varices with severe portal gastropathy. A colonoscopy showed rectal varices with colopathy. He underwent two sessions of endoscopic variceal banding. He then developed progressive jaundice (peak bilirubin - 46 mg/dL) with cholestatic features three months back and was evaluated with magnetic resonance cholangiopancreatography (MRCP), which was suggestive of PCC with cholelithiasis. He underwent endoscopic biliary drainage and plastic stent placement, after which he improved symptomatically. He was then referred to us and was evaluated with a contrast-enhanced CT, which showed features suggestive of EHPVO, such as normal liver outline with the leafy left lobe of the liver due to enlarged spleen, non-visualization of the portal vein, splenic vein, and SMV; IMV, however, was patent with a diameter of 0.8 cm and IVC was normal; no intrahepatic biliary radical dilatation (IHBRD) was seen as the patient had a common bile duct (CBD) stent in situ (Figure 1). MRI of the abdomen with MRCP confirmed PCC with bilobar IHBRD with CBD of diameter 1.6 cm with stent in situ and cholelithiasis.

**Figure 2:**
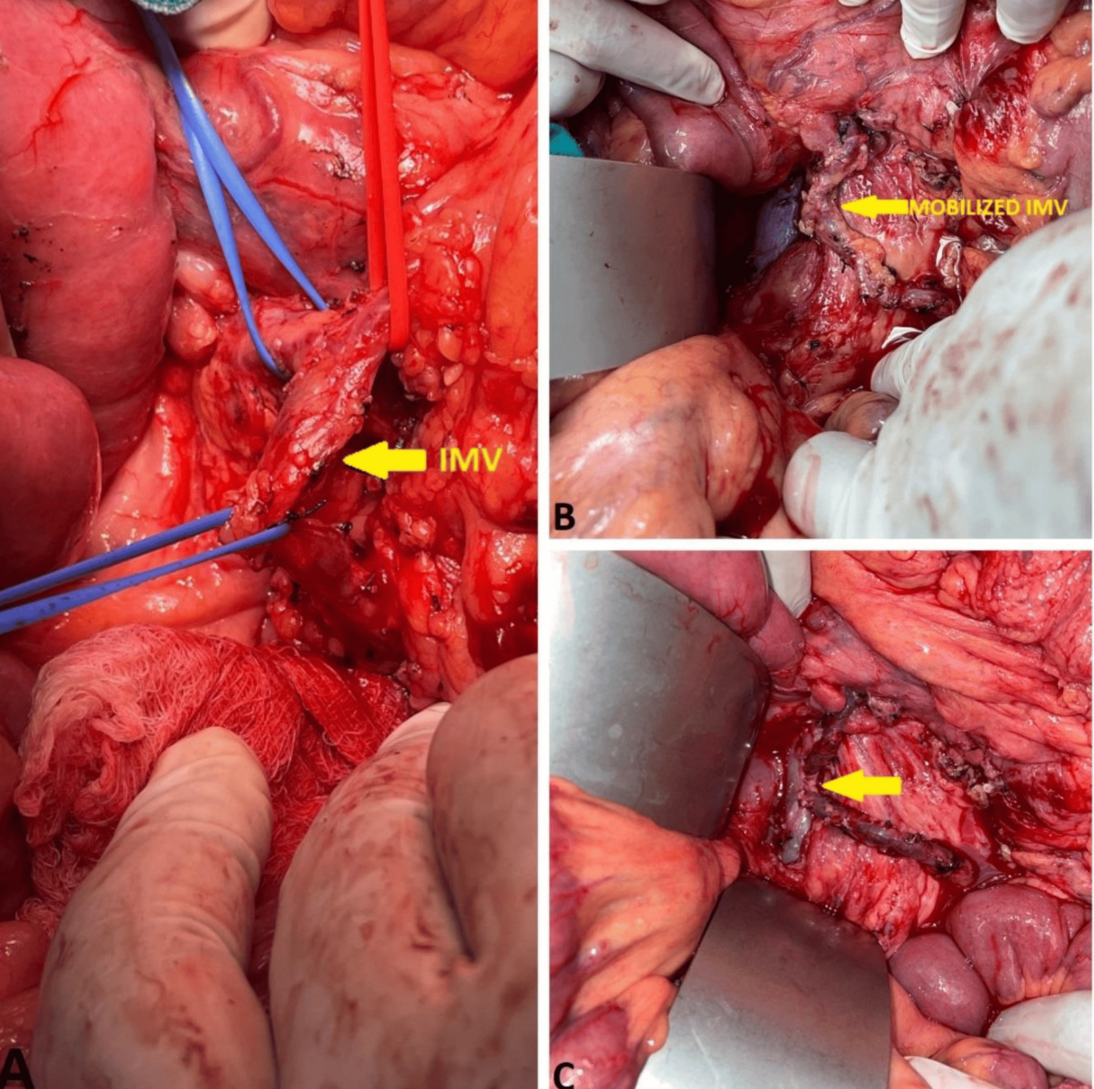
Intraoperative pictures (A) Intraoperative image showing mobilization of the inferior mesenteric vein (IMV). (B) IMV after complete mobilization. (C) Completed side-to-side IMV-caval shunt (yellow arrow).

As the patient was young and a bleeder with symptomatic PCC/colopathy, he was planned for portosystemic shunt surgery. As the imaging was suggestive of thrombosis and non-visualization of splenic, portal, and SMVs, he was provisionally planned for an unusual IMV-caval shunt. Laparotomy was performed with a modified Makuuchi incision. The splenic vein was thrombosed and sclerotic and was replaced by multiple collaterals at the splenic hilum. A splenectomy was performed. As the splenic vein could not be used for shunt, the IMV was mobilized from the IMV-splenic vein junction until the bifurcation of the aorta, and the IVC was mobilized below the left renal vein from the left side. An anterior venotomy of 1 cm was made on the IVC, and a side-to-side IMV-caval shunt was created using 6-0 Prolene (Ethicon Inc., Raritan, NJ). The shunt diameter was 1.5 cm (Figure 2).

Omental vein pressure was as follows: on opening the abdomen - 40 mmHg; after splenic artery ligation - 36 mmHg; after splenectomy but before shunt - 32 mmHg; after shunt - 23 mmHg. Postoperatively, he had chylous drain output, which was managed conservatively with dietary restrictions and modifications, and it subsided. A conventional and magnetic resonance lymphangiography was done in an attempt to localize the site of the chyle leak but with no success. He was discharged on postoperative day 18 with minimal chylous output through the drain with antiplatelet medication. The chylous output stopped spontaneously on conservative measures after one month. He is currently doing well 16 months after surgery with no further GI bleeding. The endobiliary stent has been removed, and liver function tests are normal. He is also being planned for cholecystectomy.

## Discussion

The majority of patients with EHPVO have pain related to splenomegaly, repeated infarctions, and symptomatic PCC, leading to hindrance in their daily activities. Splenectomy with PSRS is the most common surgery performed in these patients [6]. Endoscopic therapies, although less invasive, lead to direct control of bleeding, but the underlying pathophysiology of increased portal pressure remains as such, and they have drawbacks like repeated settings, follow-up, failures, cost, and logistics. One-time intervention in the form of shunt surgery is extremely beneficial in controlling symptoms and is better as it modifies the disease pathophysiology. The role of shunt surgery in modifying pathophysiology related to growth in children is well understood, as documented by Sarin et al. [7] and Menon et al. [8], but whether it is beneficial in controlling symptomatic PCC, colopathy, etc., is still debatable. PCC is asymptomatic in approximately 80% of patients with EHPVO and is symptomatic in the remaining 10-15% of patients. PCC occurs due to external compression of the CBD by the collaterals or ischemic stricture caused by PVT. Many studies have demonstrated that most strictures are due to pressure by collaterals rather than ischemic strictures, and these strictures usually resolve after shunt surgery [9]. There are two schools of thought regarding the treatment of symptomatic PCC: first, perform a shunt operation and wait for the symptoms of biliary obstruction to go away (this usually takes four to eight weeks); then, perform second stage biliary surgery if there is no resolution of symptoms despite adequate waiting time. Five of the seven patients in the report by Chaudhary et al. [9] had relief from jaundice within three to seven weeks of shunt surgery, and only two patients required second-stage hepaticojejunostomy. Another school of thought proposes endoscopic therapy (in the form of repeated or multiple stents) first for symptomatic PCC, and surgery (portosystemic shunt followed by biliary surgery) is reserved for the failures of endoscopic therapy. In both schools of thought, there is a certain role of surgery in symptomatic PCC. Other indications of surgery in EHPVO include failure of endoscopic management for GI bleed, massive splenomegaly, symptomatic hypersplenism, colopathy, a patient living in a remote area, and a rare blood group.

Our patient had symptomatic splenomegaly and hypersplenism (in the form of recurrent upper respiratory tract infections), resulting in educational and financial loss and symptomatic PCC, but did not have a shuntable vein. Thus, we decided to do this unconventional shunt. Splenectomy with devascularization may help in the control of bleeding but does not take care of PCC. Unconventional shunts using other veins are an option in the absence of a shuntable splenic vein. Literature on unconventional shunts is sparse. In a series from India [10], only 10 out of 189 patients underwent unconventional shunt over a 10-year period. The 10 unconventional shunts included eight proximal spleno-adrenal shunts, one collateral-renal shunt, and one IMV-caval shunt. Other “makeshift” shunts described in the literature are gastroepiploic vein to left renal vein, an interposition polytetrafluoroethylene (PTFE) prosthetic graft between a variceal aneurysm adjacent to the thrombosed SMV and IVC, and a direct shunt between a retro-duodenal varix and IVC [11].

Choosing the right vein for portal decompression is important, and hence, preoperative cross-sectional imaging plays an important role in planning these unconventional procedures. The IMV-caval shunt has been described in the past by Gorini et al. [12] for cirrhotics but not in EHPVO.

In the absence of a shuntable vein, endoscopic therapy was an option, but we went ahead to perform a shunt because the patient had bleeding esophageal varices, colopathy, rectal varices, and symptomatic PCC and a side-to-side IMV-caval shunt was planned based on preoperative imaging, which demonstrated thrombosed splenic and SMV and with patent IMV. In such cases, the IMV decompresses the portal venous system through the collaterals. It served the dual purpose of decompressing the portal circulation as well as rectal varices. Although the patient had morbidity due to chyle-leak, effective shunt surgery led to the resolution of PCC and removal of endobiliary stents.

## Conclusions

The management of NCPH has significantly changed following the advancements in endoscopic therapies. However, the choice of which therapy is better and which to use when is still debatable, as each has its pros and cons. Most of the time, conventional spleno-renal shunts (proximal and distal) are used, but there is a role for unusual shunts in the absence of a shuntable vein on imaging. For efficient portal decompression, surgeons performing surgery on patients with EHPVO may sometimes need to modify their preoperative strategy and use alternative unconventional shunts. Unconventional shunts can be utilized safely and efficiently in rightly selected cases with good postoperative outcomes.
